# Cholesterol Bilayer Domains in the Eye Lens Health: A Review

**DOI:** 10.1007/s12013-017-0812-7

**Published:** 2017-06-29

**Authors:** Justyna Widomska, Witold K. Subczynski, Laxman Mainali, Marija Raguz

**Affiliations:** 1 Department of Biophysics, Medical University of Lublin, Lublin, Poland; 2 Department of Biophysics, Medical College of Wisconsin, Milwaukee, USA; 3 Department of Medical Physics and Biophysics, University of Split, Split, Croatia

**Keywords:** Spin label, Membrane domains, Cholesterol, Lens cortex, Lens nucleus, Cataract

## Abstract

The most unique biochemical characteristic of the eye lens fiber cell plasma membrane is its extremely high cholesterol content, the need for which is still unclear. It is evident, however, that the disturbance of Chol homeostasis may result in damages associated with cataracts. Electron paramagnetic resonance methods allow discrimination of two types of lipid domains in model membranes overloaded with Chol, namely, phospholipid-cholesterol domains and pure Chol bilayer domains. These domains are also detected in human lens lipid membranes prepared from the total lipids extracted from lens cortices and nuclei of donors from different age groups. Independent of the age-related changes in phospholipid composition, the physical properties of phospholipid-Chol domains remain the same for all age groups and are practically identical for cortical and nuclear membranes. The presence of Chol bilayer domains in these membranes provides a buffering capacity for cholesterol concentration in the surrounding phospholipid-Chol domains, keeping it at a constant saturating level and thus keeping the physical properties of the membrane consistent with and independent of changes in phospholipid composition. It seems that the presence of Chol bilayer domains plays an integral role in the regulation of cholesterol-dependent processes in fiber cell plasm membranes and in the maintenance of fiber cell membrane homeostasis.

## Introduction

The lens has a unique structure consisting of densely packed fiber cells, most of which lose their organelles soon after they are formed [1, 2]. Thus, the plasma membrane becomes the only membranous structure of the mature fiber cells. The ability of the human lens to focus light is the result of the unique cellular and molecular architecture that eliminates light scattering and allows achievement of a high refractive index [3]. The loss of lens transparency, known as cataracts, is a disease mostly correlated with aging. It has been suggested that everyone would develop cataracts if they lived long enough [4]. The major change during cataract development is the aggregation of cytosolic proteins, crystallins, resulting in light scatter, and loss of visual acuity [5]. Some studies have shown that the plasma membrane of lens fiber cells may also be involved in the cataractogenous process [6, 7].

The most unique biochemical characteristic of the eye lens fiber cell plasma membrane is its extremely high cholesterol (Chol) content. The Chol/phospholipid (PL) molar ratio of the fiber cell membrane ranges from 1 to 2 in the cortex and can be as high as 3–4 in the lens nucleus [8]. The Chol/PL molar ratio for the typical plasma membrane is lower, <0.5 [9]. In these membranes, Chol not only saturates the PL bilayer but also leads to the formation of Chol bilayer domains (CBDs) within these membranes [10–12]. The need for high Chol content in the eye lens is not well understood. Despite this uncertainty, we know that any Chol depletion should bring negative consequences in lens microarchitecture. It is well established that inborn errors of Chol synthesis—Smith-Lemi-Optiz Syndrom, X-linked dominant chondrodysplasia punctata, mevalonic aciduria, lathosterolosis, and desmosterolosis—have negative consequences, including causing serious malformations of the brain and cataract formation [13–15]. The pathological processes underlying the developmental defects found in this group are the consequence of both accumulation of sterol precursors and Chol deficiency [14]. Another Chol deficiency linked with cataracts can be found in statin users. Therapeutic agents that block Chol synthetic pathways have cataractogenic properties in treated animal groups [16–18]. Human studies are not clear; some of them have found an increased risk of cataracts in statin users [19, 20], while others show beneficial effects of statins that decrease the risk of cataracts [21, 22] or no effects at all [23, 24]. All these studies indicate, however, that Chol plays an important physiological role in the eye lens. But, it is not clear why the Chol content becomes so high, or whether the appearance of CBDs and/or Chol crystals is harmful or beneficial for lens function [10, 11, 25–27]. The main hypothesis of this review is that the high Chol content in the fiber cell plasma membranes of the eye lens, and the presence of the CBDs, play a significant function in maintaining lens transparency and, thus, in protecting against cataract development. We believe that understanding the role of the fiber cell plasma membrane in maintaining lens transparency and, in particular, understanding the differences in the membranes of the lens fiber cells that contribute to cataracts are crucial in developing new strategies and targets for preventing/therapy of cataracts.

The effects of Chol and CBDs on the organization and properties of lens lipid membranes are illustrated by the data obtained using the electron paramagnetic resonance (EPR) spin-labeling methods. These methods provide information about the lateral organization of the lipid bilayer membranes, allowing discrimination of coexisting phases and domains as well as allowing several important membrane properties as a function of the membrane depth to be obtained. First, we will describe the unique abilities of these methods to provide guidelines for a clear understanding of the presented experimental results and our interpretation of the data.

## EPR Spin-Labeling Approaches for Discrimination And Characterization of the CBD

**Fig. 1:**
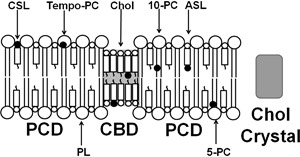
Schematic drawing showing the organization and distributions of two Chol-analog spin labels (ASL and CSL) and three examples of phospholipid-analog spin labels (Tempo-PC, 5-PC, 10-PC) in the lipid membrane with a Chol content above the Chol solubility threshold (CST). Three different Chol environments are presented: PCD (bulk phospholipid-Chol domain), CBD (Chol bilayer domain), and Chol crystal. The nitroxide moieties of spin labels are indicated by *black dots*

As indicated by Subczynski et al. (a paper in this issue), the fiber cell plasma membranes of human eye lenses belong to those with a very high Chol content. The Chol content in the cortical and nuclear membranes of donors of all ages is always high enough to form CBDs within the PL bilayer [10]. This condition ensures that the PL bilayer surrounding the CBDs is always saturated with Chol. We called this bilayer the PL-Chol domain (PCD) (Fig. 1). This unique organization of lipid bilayer membranes oversaturated with Chol provides unique opportunities in the application of EPR spin-labeling methods. In these studies, PL-analog and Chol-analog spin labels are used to mimic natural lens lipid molecules. PL-analog spin labels (like other PLs) are located only in the PCD, while Chol-analog spin labels are distributed between PCD and CBD (like Chol molecules) [11, 12, 28–30]. Figure 1 presents a schematic drawing of the distribution and orientation of spin labels. These unique distributions of spin labels ensure that (1) all membrane property profiles obtained with PL-analog spin labels describe only the properties of the PCD, without contamination from the CBD, and (2) only Chol-analog spin labels can discriminate the CBD from the surrounding PCD.

Four membrane property profiles are routinely obtained using PL-analog spin labels. Two of them, namely profiles of the order parameter [31] and of the spin-lattice relaxation rates (fluidity profiles) [32, 33], describe, respectively, the amplitude of the wobbling motion and the rate of the rotational motion of the acyl chain fragment to which the nitroxide free radical moiety (giving rise to the EPR signal) is attached. The other two, namely hydrophobicity profiles [34, 35] and oxygen transport parameter profiles [36–38] (oxygen transport parameter is proportional to the oxygen diffusion-concentration product), describe these properties in the nearby vicinity of the nitroxide moiety located at a certain depth in the membrane. (See Subczynski et al. in this issue for descriptions how these profiles are obtained from EPR measurements.)

**Fig. 2:**
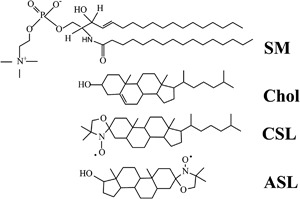
Chemical structures of sphingomyelin (SM), cholesterol (Chol), and Chol-analogue spin labels (CSL, ASL). Sphingolipids are major phospholipids of the fiber cell plasma membranes in human eye lenses

**Fig. 3:**
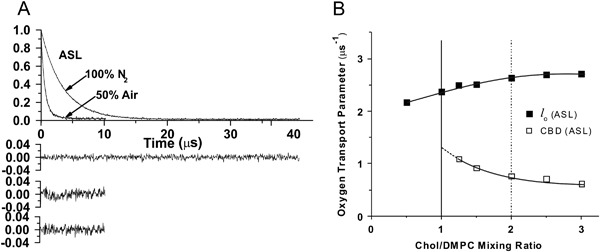
**a** Representative SR EPR signals with fitted curves and residuals (the experimental signal minus the fitted curve) for ASL in Chol/DMPC membranes with a Chol/DMPC mixing ratio of 1.5. Signals were recorded at 27 °C for samples equilibrated with 100% nitrogen and with a gas mixture of 50% nitrogen and 50% air. The single-exponential fit was satisfactory in deoxygenated (100% N_2_) membranes (*top residual*). In the presence of the hydrophobic relaxation agent (oxygen) single—exponential fit was not satisfactory (*middle residual*), while the double-exponential fit was excellent (*bottom residual*). **b** The oxygen transport parameter, calculated on the basis of eqs. (1) and (2) for ASL in the Chol/DMPC membrane is plotted as a function of the Chol/DMPC mixing ratio. The *vertical solid line* (Chol/DMPC = 1) indicates the Chol saturation threshold, and the *vertical broken line* (Chol/DMPC = 2) indicates the CST in the DMPC membrane. Figure is reproduced from reference [41], Copyright 2013, with permission from American Chemical Society

**Fig. 4:**
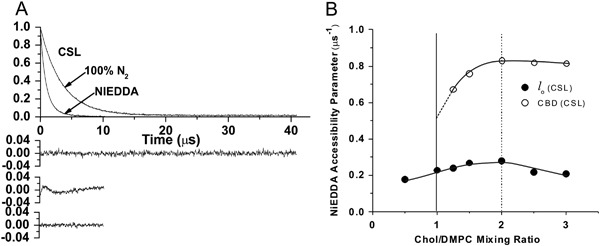
**a** Representative SR EPR signals with fitted curves and residuals (the experimental signal minus the fitted curve) for CSL in Chol/DMPC membranes with a Chol/DMPC mixing ratio of 1.5. Signals were recorded at 27 °C for samples equilibrated with 100% nitrogen. One sample contained 20 mM NiEDDA. The single-exponential fit was satisfactory without the presence water-soluble relaxation agent (NiEDDA) (*top residual*). In the presence of NiEDDA, the single-exponential fit was not satisfactory (*middle residual*), while the double-exponential fit was excellent (*bottom residual*). **b** The NiEDDA accessibility parameter, calculated on the basis of eqs. (3) and (4) for CSL in the Chol/DMPC membrane is plotted as a function of the Chol/DMPC mixing ratio. The *vertical solid line* (Chol/DMPC = 1) indicates the Chol saturation threshold, and the *vertical broken line* (Chol/DMPC = 2) indicates the CST in the DMPC membrane. Figure is reproduced from reference [41], Copyright 2013, with permission from American Chemical Society

To discriminate the CBD with Chol-analog spin labels, two dual-probe saturation-recovery (SR) EPR spin-labeling methods are applied. One method, called the discrimination by oxygen transport method [39, 40], uses the Chol-analog ASL (see Fig. 2 for ASL molecular structure), which has the nitroxide moiety located in the membrane center, and the hydrophobic relaxation agent (molecular oxygen). The second method uses the Chol-analog CSL (see Fig. 2 for CSL molecular structure), which has the nitroxide moiety located in the polar head-group region, and the water-soluble NiEDDA relaxation agent [41, 42]. When located in two different membrane domains, ASL and CSL alone (sample equilibrated with N_2_) cannot differentiate between CBD and PCD, giving indistinguishable conventional EPR spectra for these two domains [43]. Also, spin-lattice relaxation times (*T*_1_(N_2_)) are similar and are manifested in the SR EPR signal that can be fitted satisfactorily with a single exponential curve. However, differences in the lipid packing in these domains affect the partitioning and diffusion of relaxation agents in these domains; these differences are easily detected by observing the different *T*_1_s from these spin labels in the presence of relaxation agents. Observed SR signals can be fitted satisfactorily only with the double exponentials (see Figs. 3a, 4a). *T*_1_s of the double-exponential curves were assigned to the bulk PCD (*T*_1_(Air, PCD) and *T*_1_(20 mM NiEDDA, PCD)) and the CBD (*T*_1_(Air, CBD) and *T*_1_(20 mM NiEDDA, CBD)). Thus, these methods not only allow discrimination of coexisting CBDs and PCDs but also characterize them by the values of their oxygen transport parameter (obtained with ASL) [28] and NiEDDA accessibility parameter (obtained with CSL) [41–43]:

1 $$	{\rm{Oxygen}}\,{\rm{transport}}\,{\rm{parameter}}\left( {{\rm{PCD}}} \right) \\ 	= T_1^{ - 1}\left( {{\rm{Air}},{\rm{PCD}}} \right) - T_1^{ - 1}\left( {{N_2}} \right)$$

2 $$	{\rm{Oxygen}}\,{\rm{transport}}\,{\rm{parameter}}\left( {{\rm{CBD}}} \right) \\ 	= T_1^{ - 1}\left( {{\rm{Air}},\,{\rm{CBD}}} \right) - T_1^{ - 1}\left( {{N_2}} \right)$$

3 $$	{\rm{NiEDDA}}\,{\rm{accessibility}}\,{\rm{parameter}}\left( {{\rm{PCD}}} \right) \\ 	= T_1^{ - 1}\left( {20\,{\rm{mM}}\,{\rm{NiEDDA}},\,{\rm{PCD}}} \right) - T_1^{ - 1}\left( {{N_2}} \right)$$

4 $$	{\rm{NiEDDA}}\,{\rm{accessibility}}\,{\rm{parameter}}\left( {{\rm{CBD}}} \right) \\ 	= T_1^{ - 1}\left( {20\,{\rm{mM}}\,{\rm{NiEDDA}},\,{\rm{CBD}}} \right) - T_1^{ - 1}\left( {{N_2}} \right)$$

Both methods (with hydrophobic oxygen and water-soluble NiEDDA relaxation agents) provide complementary information about the lateral organization and domain properties of the membrane.

## CBD is Formed at a High Chol Content

The upper limit for Chol incorporation into the PL bilayer is called the Chol solubility threshold (CST). Above that Chol content, Chol monohydrate crystals are formed outside the lipid bilayer [41, 44]. Analogous with CST, the Chol concentration at which CBDs begin to form is called the Chol saturation threshold. The PL composition of the membrane surrounding the CBDs determines both the Chol saturation threshold and the CST. CSTs for phosphatidylserine (PS), phosphatidylethanolamine (PE), phosphatidylcholine (PC), and sphingomyelin membranes are 33, 50, 66, and 66 mol%, respectively [44–47]. It was shown that CBDs start to form at a Chol concentration significantly lower than the CST [10, 11, 41]. The results, presented in Figs. 3b, 4b, demonstrate this for DMPC membranes, thus showing that two domains—namely the CBD and the PCD—were detected at a Chol content greater than 50 mol% [41]. This is significantly lower than the Chol content of 66 mol%, which is the CST for the DMPC membrane [44]. The CBD can be discriminated using molecular oxygen (Fig. 3 and equs. (1) and (2)) or NiEDDA (Fig. 4 and eqs. (3) and (4)) as relaxation agents. Results show that formation of CBDs precedes formation of Chol crystals in both simple model membranes [41] and lens lipid membranes [10–12]. Thus, in lens lipid membranes of donors aged 0–40 years, with PLs containing significant amounts of PS and PE, CBDs can be observed at a Chol/PL molar ratio less than one.

The existence of the pure Chol bilayer domain in membranes oversaturated with Chol was proposed earlier based on X-ray diffraction measurements and the observed 34 Å diffraction pattern in membrane preparations [26, 27, 48]. The same 34 Å pattern was observed for pure anhydrous Chol crystals [44]. Because of these similarities, the observed structure (depictured as a pure Chol bilayer) was named the “cholesterol crystalline domain.” An X-ray diffraction analysis indicated that the cholesterol crystalline domain is formed by anhydrous Chol molecules with the structure as rigid as in Chol crystals. The existence of the pure Chol bilayers in membranes oversaturated with Chol was confirmed by the EPR spin-labeling method. However, because of the highly fluid structure of pure Chol bilayers discriminated by the EPR spin-labeling method, this domain was named “cholesterol bilayer domain (CBD)” [42]. ASL and CSL, when located in the PCD and CBD, give very similar conventional EPR spectra, indicating similar order and motion of Chol molecules [43]. The highly dynamic structure of the CBD was confirmed by the molecular dynamics simulation [49, 50]. Also, both the EPR spin-labeling method and molecular dynamics simulation indicated that the –OH group of Chol in the pure Chol bilayer is highly accessible to water molecules, and, thus, this bilayer cannot be formed by anhydrous Chol molecules. All these differences between the cholesterol crystalline domain and the CBD are discussed in [41]. We think these discrepancies came from the method used to discriminate membrane domains and from the preparation of the lipid bilayers. For X-ray diffraction measurements, membranes were prepared using the film deposition method, which creates artifact formation of anhydrous Chol crystals during liposome preparation [44]. Also, a 34 Å X-ray diffraction pattern can be observed only for stacks of Chol bilayers (like in Chol crystals) and not for randomly located (not stacked) pure Chol domains. To avoid these problems, the rapid solvent exchange method [44, 51, 52] was used for membrane preparation in the EPR spin-labeling experiments. We would like to conclude that the CBD [41–43] is not the same as the cholesterol crystalline domain detected using X-ray diffraction [26, 27, 48]. In our opinion, the latter is not a membrane domain of pure Chol bilayer; rather, it comprises artifacts from the formation of small anhydrous Chol crystals during liposome preparations. We also direct readers to the review by Epand et al. [53], where various preparations of Chol/PL mixtures are described and different Chol domains are indicated.

Gibbs’ phase rule prohibits treating the CBD as a new phase in Chol/PL mixtures. (See phase diagram in Subczynski et al. in this issue, and more explanation in [42].) CBDs are domains supported by the surrounding PL bilayer saturated with Chol, which makes the liquid-ordered phase of PL a structured liquid-ordered phase (or, using different terminology, a dispersed phase). Properties of the CBD (oxygen transport parameter and NiEDDA accessibility parameter) change when the Chol content increases from 50 to 66 mol% (Figs. 3b, 4b). We interpret these results as the influence of the interface, where the exchange of Chol molecules with the surrounding PL bilayer effectively affects the properties of the CBD. The increased Chol content increases the size of the CBD and decreases the effects of the interface. We also answered the questions: Is the CBD formed separately and independently in each leaflet of the bilayer? Or does it form a real bilayer as shown in Fig. 1? The results of our measurements using fluorescent-labeled PL and Chol molecules confirm that the CBD forms the bilayer structure [54].

## CBD Forms a Buffer for Chol Concentration in the Surrounding Lipid Bilayer

As follows from the phase diagram (presented in Fig. 1 in Subczynski et al. in this issue), the Chol saturation threshold for the DMPC membranes is at 50 mol%. Above this concentration, the excess Chol forms CBDs. In other PL membranes, Chol in excess of the Chol saturation threshold forms CBDs, and the remaining Chol saturates the PL bilayer. Thus, the presence of CBDs ensures that the surrounding PL bilayer is saturated with Chol. Formation of Chol crystals does not change this condition because the crystals are formed outside the membrane, possibly as a result of the collapse of CBDs that are too large. (See [55] for a schematic explanation.) Our results with lipid bilayers made from different PLs and different PL mixtures saturated with Chol indicate that profiles of the measured membrane properties (order of acyl chain, membrane fluidity, hydrophobicity, and oxygen diffusion-concentration product) are very similar (practically identical) independent of PL composition [10–12, 29, 56, 57]. Profiles for membranes with a saturating amount of Chol differ drastically from profiles across membranes without Chol [32–34, 36, 58]. Additionally, profiles for membranes without Chol depend on PL species or their mixtures. All of these allow us to conclude that *the saturating Chol content in PL membranes keeps the bulk physical properties of these membranes consistent with and independent of changes in PL composition*. We can extend this conclusion by stating that *the CBD provides a buffering capacity for Chol concentration in the surrounding phospholipid bilayer*.

**Fig. 5:**
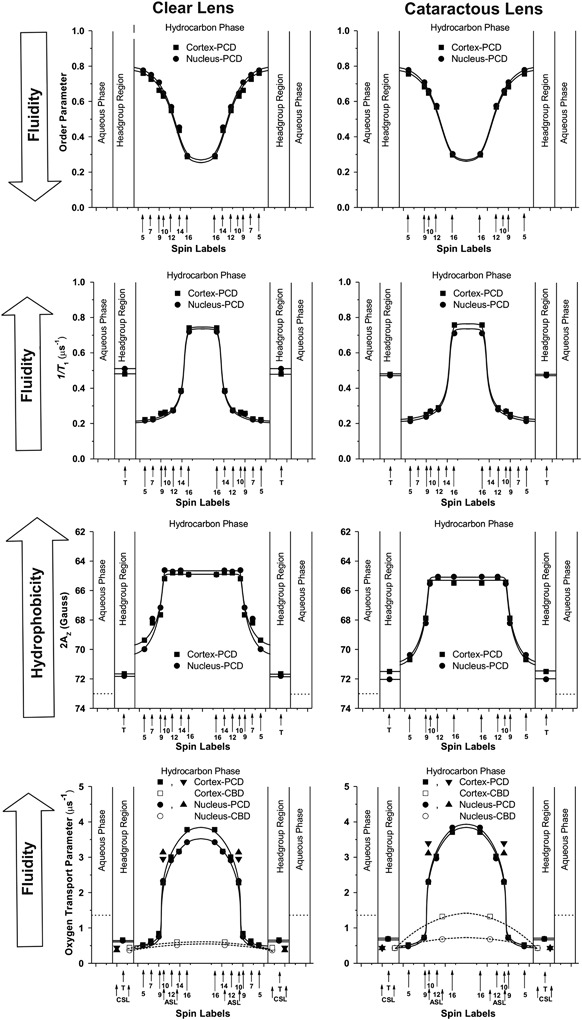
Profiles of the order parameter, fluidity (1/*T*_1_), hydrophobicity (2*A*_Z_), and the oxygen transport parameter across the PL—Chol domain (PCD) of cortical and nuclear lens lipid membranes of clear (*left column*) and cataractous (*right column*) human lenses of 61–70-year-old donors obtained at 37 °C with PL-analog spin labels. Profiles of the oxygen transport parameter across the CBD of cortical and nuclear lens lipid membranes obtained with Chol-analog spin labels are marked by *dashed lines* (*bottom profiles*). Figure is reproduced from reference [11], Copyright 2014, with permission from Springer

The effect of the saturating amount of Chol on profiles of different membrane properties can be summarize as follows: (1) Chol strongly orders PL acyl chains, which is in contrast to the low order of membranes without Chol. An ordering effect of Chol in fluid-phase membranes is observed at all depths from the membrane surface to the membrane center. (2) *T*_1_^−1^ profiles (profiles of the spin-lattice relaxation rate), which we call “fluidity profiles,” for membranes saturated with Chol (when compared with fluidity profiles without Chol) reveal that Chol has a rigidifying effect only to the depth occupied by the rigid steroid-ring structure and a fluidizing effect at deeper locations. These effects cannot be differentiated by profiles of the order parameter. Profiles of (3) hydrophobicity and (4) the oxygen transport parameter in membranes saturated with Chol have a characteristic rectangular shape with an abrupt change between the C9 and C10 positions (Fig. 5), which is approximately where the rigid steroid-ring structure of Chol reaches into the membrane. At this position, hydrophobicity increases from the level of methanol to that of hexane, and the oxygen transport parameter increases by a factor of ~3 (from the level observed in gel-phase membranes to that observed in fluid-phase membranes). These profiles are bell-shaped in phospholipid membranes without Chol.

## CBD is Observed in all Human Lens Lipid Membranes

Chol content in the human eye fiber cell plasma membranes increases with age and with the depth in the lens (and is lower in cortical than in nuclear membranes). However, these Chol contents are always high enough to saturate the PL bilayers of these membranes. This is evidenced from results (presented in Fig. 7 by Subczynski et al. in this issue) that show the CBD is always present in human lens lipid membranes prepared from the total lipids extracted from clear lens cortices and the nuclei of donors aged 0–70 years. This significant finding allowed us to extend conclusions made for simple model membranes to human lens lipid membranes, namely, that *the saturating content of Chol keeps the physical properties of the PL bilayer portion of lens lipid membranes constant with and independent of changes in the PL composition that occur with age*. Thus, properties of lens lipid membranes, both in the lens cortex and nucleus, are not determined by the PL composition, which changes drastically with the age of the donor. Drastic changes in the PL composition should exert stress on integral membrane proteins and alter their functions. This should create significant problems for fiber cell membranes because of the lack of lens protein turnover [59, 60]. It should be pointed out that fiber cells are the longest living cells in the human organism. It seems that evolution solved this problem by always keeping these membranes at a saturating Chol content (with the help of CBDs), thus ensuring that the physical properties of the lipid bilayers surrounding integral proteins are always the same, independent of age-related changes in the PL composition.

Additionally, the saturating Chol content in the plasma membrane, which—together with the cytoskeleton—forms the only supramolecular structure of the mature fiber cells, helps to create and maintain high hydrophobicity and rigidity barriers for the nonspecific permeation of polar and nonpolar molecules [57]. Thus, the transport of these molecules can be tightly controlled by integral membrane proteins to maintain appropriate conditions for the cytoplasm and the lens as a whole. The lens is an avascular, multicellular organ in which cells are coupled by a network of channels and gap junctions. Mathias et al. [61] suggested that the lens generates its own microcirculatory system, with ions and water flow directed inward at the lens poles and outward at the equator. *The unique lens circulation is secured by the high Chol content, which ensures the high hydrophobicity of the lipid bilayer portion of fiber cell membranes and the lens homeostasis*.

Whereas the physical properties of PCDs remain the same for all age groups, the properties of CBDs change significantly with the age of the donor. We think that these changes are related to the size of the CBD, which increases with the age of the donor and is mainly determined by the Chol content in the membrane [10]. It seems that *the balance between age-related changes in membrane PL composition and Chol content plays an integral role in the regulation of Chol-dependent processes in fiber cell membranes and the maintenance of fiber cell membrane homeostasis*. In that delicate balance, the PL composition determines the Chol concentration at which CBDs are formed. In humans, this delicate balance between age-related changes in membrane PL composition and Chol content delays the formation of CBDs and Chol crystals.

## CBD Enhances the Barrier for Oxygen Transport into the Lens Interior

To ensure lens transparency and prevent cataract development, the oxygen concentration in the lens, especially in the lens nucleus, has to remain at a very low level. Any oxygen concentration disturbance inside the lens leads to cataract development. A disturbance can be an acute increase in oxygen concentration after vitrectomy or hyperbaric treatment [62–64], or more chronic oxygen exposure during aging (for example, after age-related changes in vitreous [65]). The lens is well prepared to avoid changing oxygen levels. The lens is avascular, and oxygen has to diffuse into the lens interior from the surrounding environment with an already very low oxygen partial pressure of ~3 mmHg in the lens anterior and ~9 mmHg in the lens posterior [66–69]. Additionally, oxygen has to be consumed within the lens; otherwise, its concentration will be the same as outside the lens. It was shown [70] that about 90% of oxygen entering the human lens is consumed by mitochondria located in the not-yet-matured, most superficial layers of fiber cells in the lens cortex. It is postulated that nonmitochondrial oxygen consumption by the ascorbate-dependent [68] or glutathione-dependent oxygen consumption reactions [65] removes the remaining oxygen from the lens interior. A hypothetical high barrier to oxygen permeation located at plasma membranes of fiber cells should help to maintain a low oxygen partial pressure in the lens nucleus, even at a very low oxygen consumption rate in this region.

Oxygen must pass thousands of fiber cell layers on its way from the lens surface to its center. Assuming the above-indicated partial pressure of oxygen at the lens surface, 90% oxygen consumption by mitochondria, and ~3 000 fiber cell layers (with mitochondria located in the first 500 layers), we determined the oxygen concentration difference across one cell layer, which ensures zero oxygen concentration in the lens center, of ~0.1 and 0.3 nM for oxygen transport from the anterior and posterior surface, respectively. (1 mmHg relates to the oxygen concentration of ~1.5 µM.) Can fiber cell membranes (on each side of the fiber cell layer) and CBDs help to maintain the oxygen concentration difference? Our results indicate that the permeability coefficient for oxygen across PL membranes saturated with Chol is significantly lower than that across membranes without Chol, and the presence of CBDs ensures that fiber cell plasma membranes are saturated with Chol. The typical value for this permeability is 54 cm/s for the PL domain in both cortical and nuclear membranes. (A water layer of the same thickness as the PL domain has a permeability of 72 cm/s). Additionally, the permeability coefficient for oxygen across the CBD is even lower, with values of 40 and 32 cm/s in cortical and nuclear lens lipid membranes, respectively. (The permeability coefficient for oxygen across the water layer of the same thickness as the CBD is 89 cm/s [12]). These data strongly suggest that the major permeability barrier for oxygen transport into the lens interior in the lipid bilayer portion of the fiber cell plasma membrane is located at the CBD. This should help to maintain a low oxygen concentration in the lens interior, especially in the human lens nucleus, where the Chol/PL mole ratio is as high as 4 [8] and the CBD should occupy a significant part of the membrane surface. Interestingly, age-related changes in the lipid composition of the human lens [8, 71–74] indicate that the resistance of the fiber cell plasma membrane to oxygen permeation should increase with age and should be greater in the lens nucleus than in the lens cortex. (See also the discussions in [28–30, 75].) We should mention here that aged fiber cell membranes are loaded with integral proteins (mainly connexins, which make gap junctions, and aquaporins, which form water channels) [76, 77]. In these membranes, these proteins form domains, arrays, and other structures [78–81] that affect the organization of the lipid bilayer, forming additional barriers for oxygen permeation. All of these effects on oxygen permeation are summarized in [56], and our final conclusion follows: *In clear lenses, age-related changes in the lens lipid and protein composition and organization are orchestrated in a way that increases the fiber cell plasma membrane’s resistance to oxygen permeation, thus helping to maintain lens transparency and protecting against cataract formation*.

## CBD and Chol in Cataractous Lenses

**Fig. 6:**
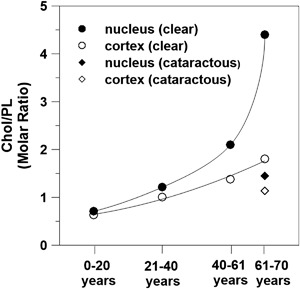
Chol content (expressed as the Chol/PL molar ratio) in cortical and nuclear lens lipid membranes of clear eye lenses from human donors of different age groups. Data obtained for cataractous eye lenses for the matched age group of 61–70 year olds are included. In all these lens lipid membranes, the CBDs were detected [10]

In this section, we compare properties of cataractous and clear lens lipid membranes of donors from matched age groups (61–70 year olds) [11]. The results presented in Fig. 5 are based on measurements for cortical and nuclear lens lipid membranes of the respective pooled samples from 30 clear and 12 cataractous human lenses. CBDs are present in all membranes, ensuring that the bulk PL bilayer is saturated with Chol. Chol content (expressed as the Chol/PL molar ratio) was evaluated for all membranes; values of 1.8 and 4.4 were found for the cortex and nucleus of clear lenses, respectively, and values of 1.14 and 1.45 were found for the cortex and nucleus of cataractous lenses, respectively. These data, together with cumulative data for age-related changes of Chol content in the cortical and nuclear membranes of clear human lenses, are presented in Fig. 6. These data indicate that age-matched cataractous lenses contain less Chol. This difference is especially pronounced for nuclear membranes. The high Chol content in clear nuclear membranes induced formation of Chol crystals (which are not observed in cataractous nuclear membranes). These results are in agreement with other reports that Chol content in cataractous lenses is lower than in clear lenses [48]. However, some papers indicate opposite trends [82, 83].

As indicated above, the saturating amount of Chol ensures that the physical properties of the lens lipid cataractous membranes (their PL bilayer portion) are the same as those of clear membranes. This is exemplified by the profiles presented in Fig. 5. Differences are only observed for profiles of the oxygen transport parameter across CBDs formed in the cortical and nuclear membranes from the two donor’s groups. These differences are easily explained by the different Chol content in these membranes and the different size of the CBDs (See Section 3). Thus, these results indicate that, in all membranes, CBDs help to maintain fiber cell membrane homeostasis and proper functioning of membrane integral proteins. These results also indicate that the presence of Chol crystals is not harmful and does not compromise lens transparency. (See Section 4.5 in Subczynski et al. in this issue for more explanation.) Our final statement based on these results follows: *The high Chol content, formation of CBDs, and formation of Chol crystals should not be regarded as major predispositions for the development of age-related cataracts*.

## Final Discussion

Finally, we would like to discuss whether the high Chol content and the presence of CBDs in the fiber cell plasma membrane are beneficial or harmful in maintaining lens transparency and other lens functions, including the focusing of light on the retina. Presented data focus on the positive aspects of the high Chol and presence of CBDs on lens health. Final statements are noted above in italics and are not repeated in this section. The age-related changes in lipid composition during the maturation of fiber cells, which include increases in the sphingolipid content, the saturation of PL acyl chains, and the Chol content, may also contribute to the increased protection against oxidative damage, which is the major cause of cataract development. The sphingolipid content of the plasma membranes of the mature human eye lens fiber cells is extremely high (~66 mol% of total PLs [84]). The presence of CBDs in these membranes ensures that the PL bilayer of the human fiber cell membranes is always saturated with Chol, which strongly decreases accessibility of oxygen into the bilayer (decreasing oxygen solubility and the oxygen diffusion coefficient). It should be noted that Chol forms very tight complexes with sphingolipid molecules [47, 85], maximizing the protective effects against oxidative stress and lipid oxidation. Possibly, in cataractous lenses, this protective mechanism is not working properly. Girao et al. [86] reported that cataractous lenses contain quantifiable amounts of Chol oxides, whereas Chol oxides were not detected in clear lenses.

The increased sphingolipid content in fiber cell plasma membranes in the presence of CBDs may also contribute to the increased stiffness of the lens and the development of presbyopia. Mechanical properties (elastic properties) of the lipid bilayer are directly related to another membrane parameter, namely lateral pressure, or, more accurately, to the lateral pressure distribution (profile) in the lipid bilayer [87, 88]. It has been shown that the mechanical rigidity of the PL bilayers increases with Chol content [89, 90], and this effect is much more pronounced for sphingolipids than for other PLs presented in fiber cell membranes [90]. The age-related stiffness of the lens is most often associated with the age-related changes that occur within the cytosolic proteins in the lens [91, 92]. However, because the total stiffness of the lens depends on the rigidity of thousands of individual membranes, the membrane contribution should also be considered as a significant factor making the lens harder and less elastic over time. These changes certainly are harmful and compromise the focusing property of the lens.

Please note that the hypotheses presented in this review, regarding the purported functions of Chol and CBDs, are based on measurements for lens lipid membranes and model lipid membranes, i.e., membranes without a protein component. Intact fiber cell membranes are loaded with integral proteins. Similar to the organization of lipids, the organization of these integral proteins (mainly aquaporins and conexins) change significantly with age, including the formation of domains, arrays, and other structures [78–81]. The amount of proteins and their packing in fiber cell membranes might modify the presented lipid physical properties and organization described for lens lipid membranes. However, to assess the contributions of integral proteins into the properties and organization of the lipid bilayer portion in intact fiber cells, first we must clearly understand the organization and properties of membranes prepared from the total lipids extracted from these membranes.
